# Aberrant expansion of CXCR5^+ ^memory CD4 T cells in patients with systemic lupus erythematosus

**DOI:** 10.1186/ar4636

**Published:** 2014-09-18

**Authors:** Jin-Young Choi, Sang Taek Kim, Insoo Kang, Joe Craft

**Affiliations:** 1 Yale School of Medicine, New Haven, CT, USA

## Background

Autoreactive B cells in SLE undergo autoantigen selection, suggesting a requirement for germinal center follicular helper T (Tfh) cells in their maturation. However, evidence for dysregulation of Tfh cells in SLE and their potential contribution to disease remains unclear. Recently, blood CXCR5^+ ^CD4 T cells, a heterogeneous pool consisting of functionally distinct Th1-like, Th2-like, and Th17-like subsets, have been proposed to be the circulating (blood) memory Tfh cells. We hypothesized that expanded CXCR5^+ ^memory cells in the blood of human lupus patients promote B-cell helper function reflecting the abnormal T-B-cell responses in secondary lymphoid organs.

## Methods and results

We characterized such cells in SLE patients by flow cytometry and T-B coculture studies. SLE patients had significant expansion of CXCR5^+^ICOS^hi^PD-1^hi ^CD4 T cells compared with controls. Such cells were Bcl6^-^, but robustly expressed IL-21 with a portion Ki-67^+^, indicating their functional activity. The blood cells were capable of providing blood-born memory B cells with survival and differentiation signals to secrete isotype switched Igs, including antinuclear antibodies.

## Conclusions

We speculate that therapies which alter T-B collaboration in lupus will abrogate their expansion, accompanied by reduced autoantibody titers and improved disease activity in SLE patients. Our results suggest that aberrant T-B collaboration in lupus is critical to disease pathogenesis and its blockade is likely to be important therapeutically.

